# Population attenuation in zooplankton communities during transoceanic transfer in ballast water

**DOI:** 10.1002/ece3.2349

**Published:** 2016-08-02

**Authors:** Sara Ghabooli, Aibin Zhan, Esteban Paolucci, Marco R. Hernandez, Elizabeta Briski, Melania E. Cristescu, Hugh J. MacIsaac

**Affiliations:** ^1^ Great Lakes Institute for Environmental ResearchUniversity of WindsorWindsorOntarioN9B 3P4Canada; ^2^ Research Center for Eco‐Environmental SciencesChinese Academy of Sciences18 Shuangqing RoadHaidian DistrictBeijing100085China; ^3^ Museo Argentino de Ciencias Naturales “Bernardino Rivadavia” and Consejo Nacional de Investigaciones Científicas y TécnicasBuenos AiresArgentina; ^4^ GEOMARHelmholtz Centre for Ocean Research KielDüsternbrooker Weg 20D‐24105KielGermany; ^5^ Biology DepartmentMcGill UniversityMontrealQuebecH3A 1B1Canada

**Keywords:** ballast water, biological invasion, genetic diversity, invasive species, Ion torrent PGM, next‐generation sequencing, nonindigenous species, zooplankton

## Abstract

Successful biological invasion requires introduction of a viable population of a nonindigenous species (NIS). Rarely have ecologists assessed changes in populations while entrained in invasion pathways. Here, we investigate how zooplankton communities resident in ballast water change during transoceanic voyages. We used next‐generation sequencing technology to sequence a nuclear small subunit ribosomal DNA fragment of zooplankton from ballast water during initial, middle, and final segments as a vessel transited between Canada and Brazil. Operational taxonomic unit (OTU) diversity decreased as voyage duration increased, indicating loss of community‐based genetic diversity and development of bottlenecks for zooplankton taxa prior to discharge of ballast water. On average, we observed 47, 26, and 24 OTUs in initial, middle, and final samples, respectively. Moreover, a comparison of genetic diversity within taxa indicated likely attenuation of OTUs in final relative to initial samples. Abundance of the most common taxa (copepods) declined in all final relative to initial samples. Some taxa (e.g., Copepoda) were represented by a high number of OTUs throughout the voyage, and thus had a high level of intraspecific genetic variation. It is not clear whether genotypes that were most successful in surviving transit in ballast water will be the most successful upon introduction to novel environments. This study highlights that population bottlenecks may be common prior to introduction of NIS to new ecosystems.

## Introduction

Biological invasions are commonplace in many habitats colonized by humans. Successful invasions are contingent upon introduction of sufficient individuals to constitute a viable population, tolerance of ambient conditions, and successful integration into the existing community (Colautti et al. 2006; Blackburn et al. 2011). These requirements must be met across an ordered series of stages from transport, introduction, establishment, and spread (Blackburn et al. 2015). Small population inocula and differences between native and introduced habitats may cause invasions to fail or trigger evolutionary changes in colonizing species (e.g., Phillips et al. 2006; Moran and Alexander 2014; Blackburn et al. 2015). Biological invasions may be viewed as examples of in situ evolution in consequence (Lee 2002; Facon et al. 2006; Barrett 2015; Colautti and Lau 2015).

A number of studies have documented successfully introduced populations with the same or higher levels of genetic diversity than putative source populations (e.g., Roman 2006; Taylor and Keller 2007; Gillis et al. 2009). Enhanced genetic diversity may result from high propagule pressure (i.e., number of introduced individuals), particularly if it involves admixis from more than one source population (Roman and Darling 2007; Muirhead et al. 2008). In seemingly rare instances, small population size may be beneficial if some of the introduced individuals carry genotypes preadapted to the novel environment (e.g., Lavergne and Molofsky 2007). More typically, however, attenuation of propagules during transportation may result in small population inocula, with population genetic bottlenecks resulting from either losses during transportation or immediately upon introduction (see Roman and Darling 2007). Loss of genetic diversity can be fatal for introduced populations if they are unable to respond to selective pressures in the new region (e.g., Dlugosch and Parker 2008; Dlugosch et al. 2015). Impoverished genetic diversity also may result from postestablishment processes, notably genetic drift and selection in the new environment (e.g., Koskinen et al. 2002; Lee et al. 2007).

Few studies have focused on dynamics that occur while nonindigenous species (NIS) are carried by the invasion pathway (Olenin et al. 2000; Ruiz et al. 2000; Wonham et al. 2001; Briski et al. 2014). This dearth of research is surprising given that principal aquatic invasion pathways such as ships’ ballast water and hull fouling each may carry dozens or more species at once (Sylvester et al. 2011; Briski et al. 2013). Wonham et al. (2001) found more than 50% loss of plankton taxa in ballast water of an ocean‐going vessel that travelled from Hadera, Israel to Baltimore, USA, during a 16‐day voyage, while Briski's et al. (2014) conceptual model of community dynamics during transportation indicates loss of 80–99% of individuals per species depending of taxonomic group during 25 days of transport in ships’ ballast tanks. The endpoint for ballast populations that have suffered severe demographic decline could be local extirpation. Examination of community dynamics during transport may help determine whether bottlenecks in NIS populations develop before and/or after introduction.

Detecting species present at very low population density can be highly problematical, although advances in genetic technologies may assist researchers in this endeavor (Jerde et al. 2011; Zhan and MacIsaac 2015). The growing use of next‐generation sequencing (NGS) is one such technology that may be employed in biodiversity studies (Hajibabaei et al. 2011; Zhan et al. 2013). For example, Zhan et al. (2013) determined that NGS could detect individual larvae or fragments down to 10^−5^% biomass contribution in plankton samples, far below traditional microscopical analysis. Here, we use NGS to assess community changes in zooplankton entrained in ballast water of vessels moving from Canada to Brazil. We assess temporal changes in zooplankton community and determine the severity of population attenuation and whether genetic bottlenecks may have resulted in consequence prior to ballast water discharge.

## Materials and Methods

**Figure 1:**
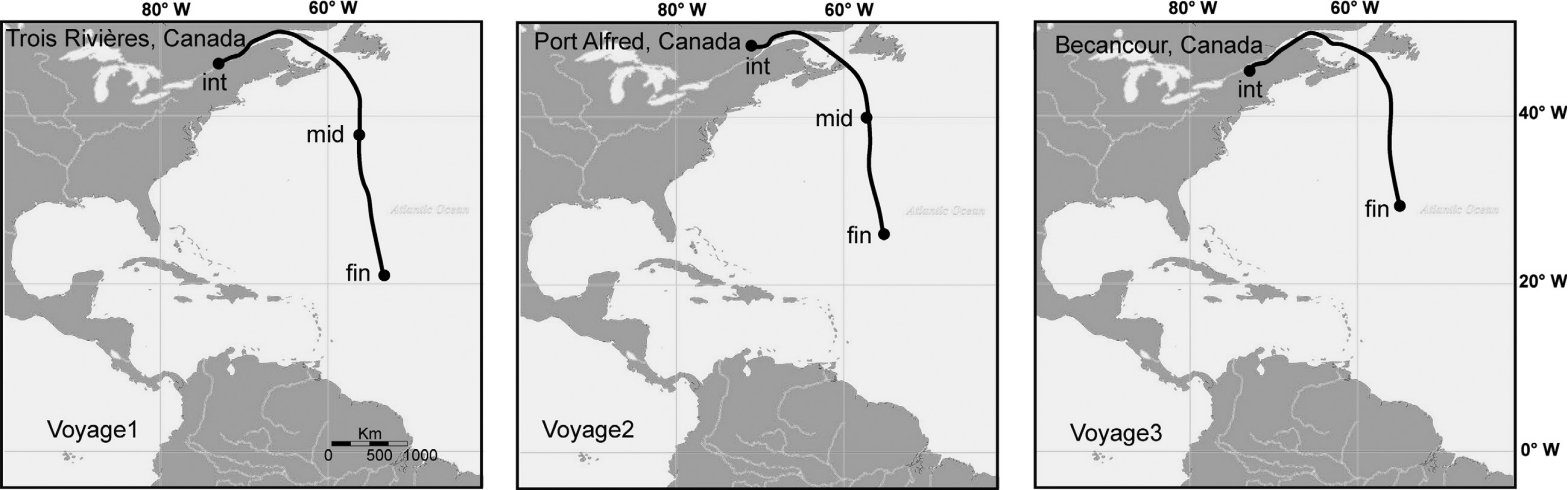
Voyage routes and the sampling locations at the initial (int), middle (mid), and final (fin) point of the experiment.

We assessed zooplankton community dynamics in a vessel moving from Canada to Brazil during voyages in July, September, and October 2012 (Fig. 1). Two ballast tanks (three tanks for the second voyage) were sampled at the beginning, middle, and prior to the end of the voyage when mandatory ballast water exchange (BWE) occurred. Middle samples were not taken in voyage three due to inclement weather. In total, 19 ballast water samples were collected during the three voyages. Equal volumes of water were pumped from three different depths in each ballast tank and combined to achieve a total sample volume of 1000 L, following which it was processed through a 35‐*μ*m plankton net. Filtered samples were transferred to 95% ethanol and stored at cool temperature on the vessel, and later processed in the lab.

### Zooplankton community genetic composition

Ethanol‐preserved samples (~60 mL) were shaken to randomize the distribution of plankton. Two replicates of 1.5 mL were taken from each preserved sample using eppendorf tubes. Tubes were centrifuged at 9279.4 g to remove ethanol. Total genomic DNA was extracted from each sample using DNeasy Blood and Tissue Kit (Qiagen Toronto, ON, Canada). Extracted DNA was PCR‐amplified using the primer pair Uni18S (5′‐AGGGCAAKYCTGGTGCCAGC‐3′)—Uni18SR (5′‐GRCGGTATCTRATCGYCTT‐3′) spanning the hypervariable V4 region of nuclear small subunit ribosomal DNA (nSSU rDNA) (Zhan et al. 2014). A 25 *μ*L PCR cocktail contained 100 ng of genomic DNA, 1 × PCR buffer, 2 mmol/L of Mg^2+^, 0.2 mmol/L of dNTPs, 0.4 *μ*mol/L of each primer, and 2U of *Taq* DNA polymerase (Genscript). PCR cycling parameters consisted of an initial denaturation step at 95°C for 5 min, followed by 25 cycles of 95°C for 30 s, 50°C for 30 s, 72°C for 90 s, and a final elongation step at 72°C for 10 min. Two PCR replicates were prepared for each sample. Samples were prepared for amplicon sequencing on an Ion Torrent Personal Genome Machine (PGM) according to the manufacturer's protocols.

Raw sequences obtained from Ion Torrent PGM were trimmed (e.g., homopolymer ≤8, maximum number of ambiguous nucleotides = 0) using the software Mothur v. 1.31.2 (Schloss et al. 2009). The UPARSE v7.0.1001 pipeline was used to remove chimeric sequences and errors/artifacts with the default settings (Edgar 2013). The resulting sequences were clustered into similarity‐based operational taxonomic units (OTUs) at a cutoff value of 3% divergence (Kunin et al. 2010; Edgar 2013). Taxonomic status of OTUs was defined by BLASTn queries against the GenBank database implemented in the pipeline Seed v.1.1.35 (Větrovsky and Baldrian 2013). OTUs with minimum query coverage of 70% and E‐value <10^−70^ were used for downstream analyses. High levels of intraspecific genetic divergence and polymorphism increase the chance of error when comparing genetic diversity of different samples (Lee 2000; Brown et al. 2015). Hence, we defined taxa at the family level to avoid uncertainty in defining intraspecific genetic diversity (Fig. S1). Analysis of variance (one‐way ANOVA) implemented in SPSS v.20 (SPSS Inc, Chicago, IL) was performed to investigate differences among average number of OTUs/sequences obtained from initial, middle, and final samples using a block design ANOVA and tanks as the blocking factor. Phylogenetic relationships of OTUs were reconstructed using neighbor‐joining (NJ) analysis in MEGA v.4 (Tamura et al. 2007).

### Zooplankton community abundance

Numerical abundance of zooplankton present in ballast samples was enumerated after taking subsamples for DNA extraction. This was carried out to evaluate the results from genetic analysis. As not all taxa were present in all samples, we focused on the most abundant taxon (i.e., Copepoda). All copepods including nauplii were counted. To estimate OTUs of the larger sampling size (i.e., more tanks) based on findings from our sampled tanks, we calculated Chao‐1, an estimator of species richness based on the number of rare species in a sample (Chao 1984; Chao and Shen 2003). Sample‐based OTUs rarefaction curves were generated to determine whether a significant difference existed given our small sample size. Chao‐1 estimates were calculated using SPADE software (Chao and Shen 2006), while rarefaction curves were generated with 5000 random iterations using ECOSIM (Gotelli and Entsminger 2006).

## Results

A total of 3,576,841 sequences were obtained from 19 samples taken from ballast tanks during the three voyages. After filtering and removing low‐quality sequences, as well as removing sequences from other groups such as bacteria and algae, 3.10% of sequences were used for downstream analyses of zooplankton community. The number of obtained OTUs varied between 12 and 64 among samples (Table 1).

**Table 1:** Operational taxonomic units (OTUs) and number of copepods recovered from three ballast tanks (A, B, and C) during three Atlantic voyages of a vessel. Each tank was sampled at the beginning, middle, and near the end of the voyage. Days refer to the time since start of the voyage when sampling was conducted

Tank	Sampling period	Days	No. of OTUs	No. of taxa (Families)	No. of copepods	No. of OTUs (copepods)
1A	Initial	0	57	23	5340	20
	Middle	4	30	10	4179	13
	Final	8	22	10	1050	10
1B	Initial	0	50	17	1,1804	17
	Middle	3	28	12	1,1231	11
	Final	7	12	7	2140	9
2A	Initial	0	35	18	4058	15
	Middle	3	18	10	3005	10
	Final	7	30	17	1431	12
2B	Initial	0	39	18	2500	18
	Middle	3	26	14	2221	17
	Final	7	26	12	896	16
2C	Initial	0	46	16	3421	24
	Middle	3	30	15	2483	16
	Final	7	42	23	1762	27
3A	Initial	0	64	34	1503	28
	Final	12	20	12	25	5
3B	Initial	0	41	25	1048	15
	Final	14	20	15	17	8

**Figure 2:**
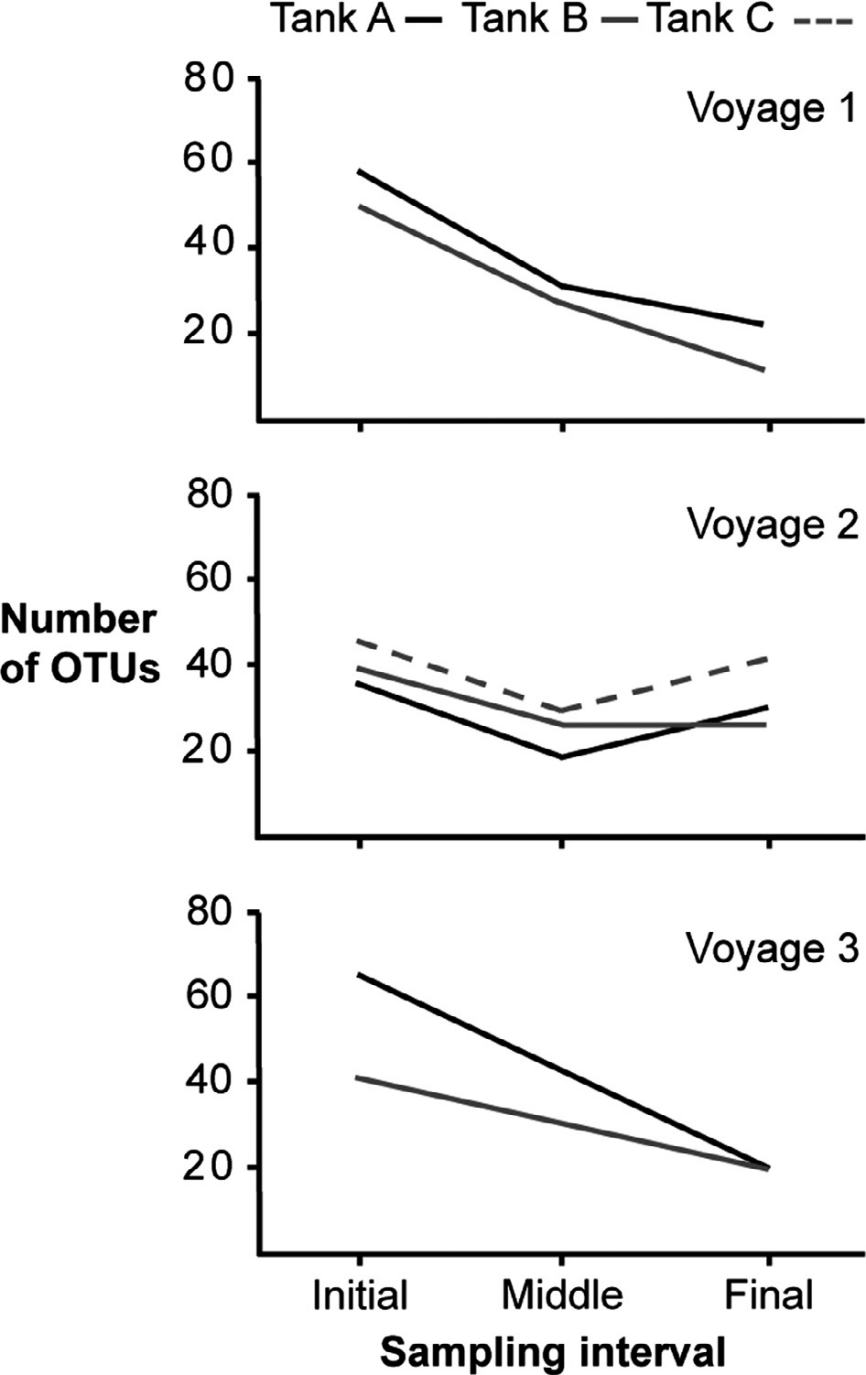
Number of OTUs (total counts) recovered from initial, middle, and final samples. Three different ballast tanks were sampled: A (black line), B (gray line), and C (dashed line). Voyage 3 was sampled only at beginning and end.

**Figure 3:**
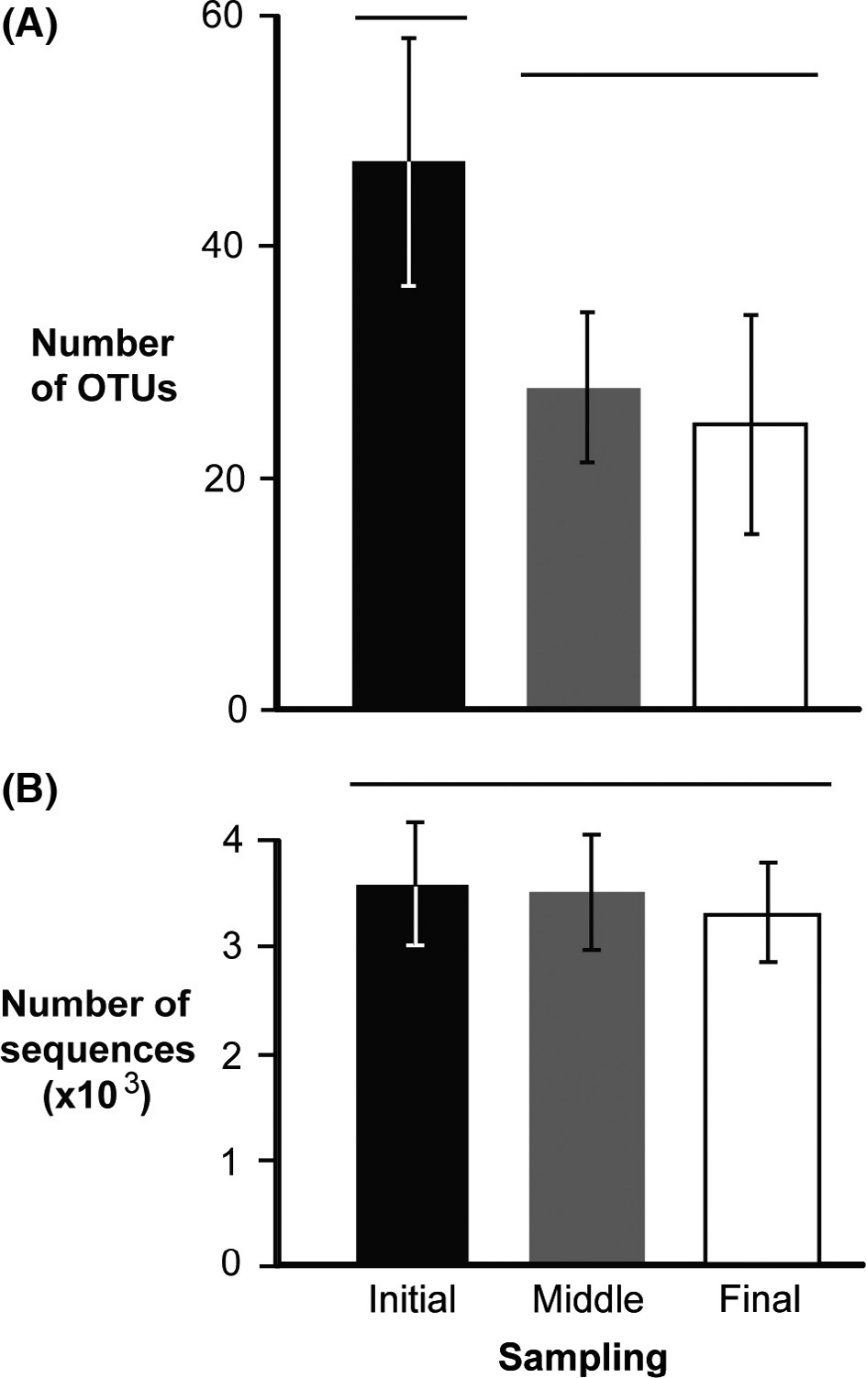
Average (±SD) number of OTUs (A) and average (±SD) number of sequences (B) obtained from all initial (black bar), middle (gray bar), and final (white bar) samples. Groups that are significantly different are not joined by the same line above the bars.

The number of OTUs decreased from the start to the end of each voyage, suggesting zooplankton die‐off in ballast tanks (Fig. 2). The mean number of OTUs recovered from initial samples of all three voyages differed significantly from that found in the middle and final samples (ANOVA, *F* = 15.17, *P *=* *0.001) (Fig. 3A), while trip differences (i.e., block effect) were not significant (*F* = 0.83, *P *=* *0.574) (Table S2). Conversely, the mean number of sequences obtained from initial, middle, and final samples did not differ significantly (ANOVA, *F* = 1.19, *P *=* *0.345), although a significant block effect was observed (*F* = 4.80, *P *=* *0.015) (Table S2). These results indicate that differences in OTU depletion rate over time were not due to the number of recovered sequences (Fig. 3B).

Voyage one exhibited the highest loss of OTUs from initial to final samples, declining by 61.4% and 76.0% in tanks 1A and 1B, respectively (Table 1). In voyage two, attenuation was less severe, with losses of 14.2%, 33.3%, and 8.6% for tanks 2A, 2B, and 2C, respectively (Table 1). A small rebound in the number of OTUs was experienced at the end of the trip in tank 2A. There were slightly more OTUs in final samples than those collected at the midpoint of the trip (Table 1). In voyage three, 68.7% and 51.2% of OTUs were lost between initial and final samples in tanks 3A and 3B, respectively (Table 1). The initial sample collected from tank 3A contained the highest number of OTUs (64) and recovered taxa (34 taxa) (Fig. 2, Table 1), while the final sample of tank 1B exhibited the lowest number of OTUs (12) and recovered only seven taxa (Fig. 2, Table 1). Some major groups such as copepods, molluscs, and protozoans appeared in all samples (Table 2). However, bryozoans, cnidarians, gastrotriches, nematodes, platyhelminthes, poriferans, and rotifers were present in only some samples (Table 2).

**Table 2:** Number of OTUs recovered from ballast tanks (A, B, and C) for three Atlantic voyages after BLASTn query against GenBank nucleotide database. Numbers indicate results for 18S marker obtained from Ion Torrent Personal Genomic Machine at the initial (int), middle (mid), and final (fin) day of the voyage. Refer Table 1 for number of days between initial, middle, and final samples

Tank	Bryozoa	Cnidaria	Copepoda	Gastrotricha	Mollusca	Nematoda	Platyhelminthes	Porifera	Protozoa	Rotifera
No. of OTUs per group (int/mid/fin)										
1A	1/1/1		20/13/10	2/0/0	18/11/8		1/0/0	1/0/0	14/5/3	
1B	1/1/0	1/1/0	18/11/9	1/0/0	21/8/2	1/0/0	1/0/0		6/7/1	
2A	2/0/1	0/1/1	15/10/12	1/0/0	3/3/3	1/0/1	1/0/0		10/4/11	2/0/1
2B	1/0/0	1/1/1	18/17/16		4/3/3	1/0/0	1/1/0		12/3/6	1/1/0
2C	1/1/0	0/0/2	24/16/27	2/0/0	5/3/3	1/0/1			12/8/8	1/1/1
No. of OTUs per group (int/fin)										
3A	1/0	1/0	28/5	2/1	4/1	2/0	2/0		20/13	4/0
3B	1/1		15/8	2/1	3/2	2/1	2/0		11/5	5/2

In voyage one, only 12 of the initial 27 taxa were present in final samples (Fig. S2). Copepods had the highest number of OTUs recovered in final samples of this voyage, representing six taxa (Fig. S2). Another six taxa were recovered (one bryozoa, two mollusca, and three protozoa) in final samples. Tetrahymenidae (Phylum: Ciliophora) was the only taxon represented by two OTUs and a single sequence in final samples of tank 1A and was not detected in previous samples of the voyage. We recovered 36 taxa from samples of voyage two, only four of which were not recovered from final samples, while 12 taxa (five copepoda, one mollusca, one cnidaria, and five protozoa) had a higher number of OTUs relative to initial samples (Fig. S3). The overall number of OTUs declined or remained the same in all major groups in this voyage, except for cnidarians which contained more OTUs in final (4) than initial samples (3) (Fig. S3). In total, 38 taxa were obtained from initial samples of voyage three, 18 of which were not present in final samples. The number of OTUs declined over time in all groups, with protozoa and copepods containing the highest number of OTUs in final samples relative to other groups (Fig. S4).

**Figure 4:**
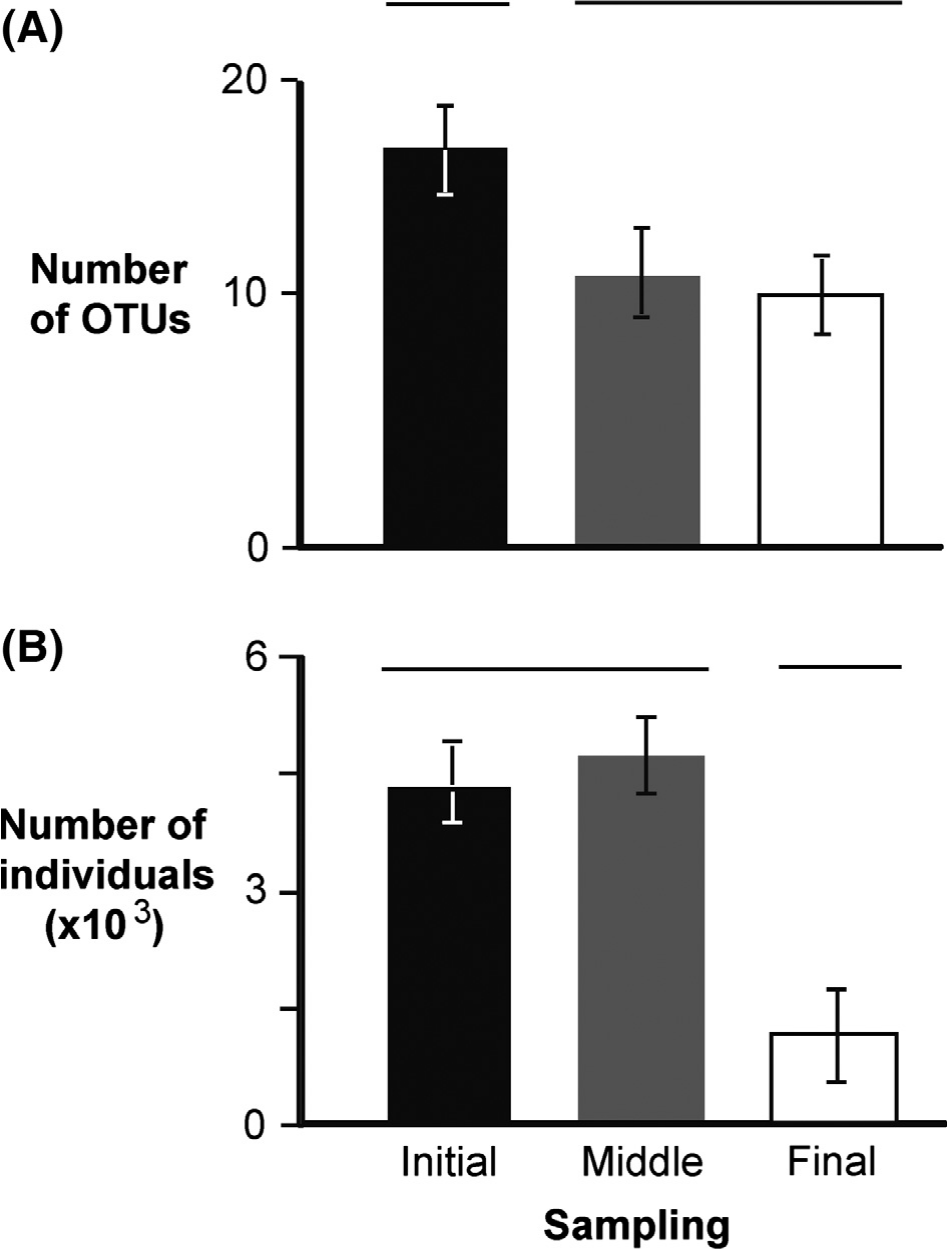
Average (±SD) number of OTUs (A) and average (±SD) number of individuals (B) for copepods obtained from all initial (black bar), middle (gray bar), and final (white bar) samples. Groups that are significantly different are not joined by the same line above the bars.

Similar to the number of OTUs, the abundance of copepods declined from the start to the end of each voyage (Fig. 4). The initial sample collected from tank 1B contained the highest number of copepods (*n* = 11804), while final sample of tank 3B had the lowest (*n* = 17) (Table 1). The highest and lowest number of copepod OTUs (*n* = 28, *n* = 5) was recovered from initial and final sample of tank 3A, respectively (Table 1). The mean number of copepods and their OTUs recovered from initial samples of all three voyages differed significantly from that found in the final samples (ANOVA, *F* = 5.02, *P *=* *0.020; *F* = 4.09, *P *=* *0.036, respectively) (Fig. 4).

## Discussion

In current study, we assessed changes in zooplankton communities in ballast water during the course of three Atlantic voyages. Our findings indicate attenuation of broad zooplankton groups during each of the voyages (Figs S2–S4, Table 1). We also demonstrate that genetic diversity is lost prior to an introduction event, although results were taxon‐specific as some species were detected for the first time toward the end of the voyage. Consistent with Wonham et al. (2001), we found that zooplankton species represented by OTUs and copepod abundance were reduced preintroduction and that not all taxa survive to the end of the voyage (Figs S2–S4, Table 1). Copepods, mollusks (veliger larvae), and protozoans were dominant among groups whose genetic diversity did not decline during voyages.

The total number of OTUs decreased along each voyage, and initial samples contained taxa that were not recovered at the end of voyage (Table 2, Figs S2–S4). Thus, our findings suggest the development of a genetic bottleneck and loss of potential genetic diversity prior to introduction. The loss of diversity is generally perceived as a significant barrier to successful establishment that must be overcome at the initial stage of an invasion (Blackburn et al. 2011). However, our results suggest that the same barrier may also occur within species.

Voyage one samples exhibited the highest loss of OTUs (76% for tank 1B) from initial to final samples (Table 1). This high loss of OTUs relative to other voyages may be due to enhanced fluctuations in temperature and salinity during the sampling period (Table S1). Temperature decreased by 5.3°C from initial samples to middle samples and then increased by 7.2°C between middle and final samples. During the same voyage, mean salinity increased in middle samples (3.1 ppt) relative to initial ones (0.1 ppt) but then decreased to final samples (0.3 ppt) (Table S1). Such fluctuations in environmental characteristics could trigger physiological shock in some taxa with adverse effects on genetic diversity in zooplankton (e.g., Cervetto et al. 1999; Zajaczkowski and Legezynska 2001).

In contrast, voyage two exhibited the lowest loss in OTU number, ranging from 8.6% to 33.3% relative to initial samples. Environmental temperature increased by 15.9°C from initial to final sample periods, while salinity decreased after initial sampling and remained relatively constant thereafter (Table S1). We observed a high loss of OTUs (>50%) for both tanks during voyage three (Table 1). This voyage was the longest trip (12 and 14 days before taking final sample for tanks 3A and 3B, respectively), which lasted for 7 days before final sampling was conducted (Table 1). Temperature of ballast water decreased by 5.1°C and salinity increased during voyage three (Table S1). Based on the above, environmental factors in ballast tanks during each voyage appear to influence the rate at which OTUs were lost or, more rarely, gained. The appearance of some taxa or an increase in their OTU number in final samples could be the result of random sampling errors (Olenin et al. 2000) or population growth (Gray and MacIsaac 2010) during the voyage, perhaps from hatching of dormant stages (Briski et al. 2010, 2011).

The total number of copepods decreased along all voyages. Voyage three—the longest trip—exhibited the highest loss of individuals at about 98%. In voyage one, more than 80% of copepods were lost in final samples. However, voyage two exhibited the lowest loss in number of copepods. A conceptual model developed by Briski et al. (2014) suggests that factors such as the length of transport and taxon‐specific survival could affect the magnitude of change in zooplankton community of ballast tanks.

A number of studies have investigated common errors associated with Ion Torrent PGM data, including erroneous insertions/deletions (i.e., indels) (Loman et al. 2012; Quail et al. 2012). Indels introduced by inaccurate flow calls appear at a rate of 1.38% in PGM data (e.g., Bragg et al. 2013). There exist a growing number of algorithms to minimize these errors for downstream analyses (Yeo et al. 2012; Flynn et al. 2015). However, much improvement is required to increase the efficiency of these methods. Effects of such errors are more pronounced when NGS data are used for polymorphism studies (Bragg et al. 2013). We used the UPARSE pipeline (Edgar 2013), which promises to produce the most accurate number of OTUs. In this method, OTUs are produced with ≤1% incorrect bases versus >3% generated by other methods (e.g., Mothur, QIIME) which tend to overestimate OTU number (Edgar 2013). Even though the UPARSE method might not represent the exact number of OTUs present in each sample, it appears to be among the most reliable methods currently available for such analyses (Edgar 2013; Flynn et al. 2015).

Results from BLAST may not be fully accurate in part due to a lack of online sequence references for particular taxonomic groups (Briski et al. 2016). Moreover, studies have shown that some groups of zooplankton—such as copepods and rotifers—form species complexes that are poorly defined taxonomically (e.g., Lee 2000; Gomez et al. 2002). We acknowledge that the number of sequences might not directly correspond to the number of propagules in ballast water (Weber and Pawlowski 2013; Flynn et al. 2015), as multiple divergent amplicons can be produced from a single individual or closely related taxa might be joined into one OTU. Therefore, our results are based upon genetic composition of the zooplankton community in the ballast water and do not fully correspond to the actual abundance of species. However, results from the abundance of copepods were in agreement with the genetic composition of zooplankton found in our ballast tanks.

In conclusion, this study highlights the possible creation of population bottlenecks prior to introduction of NIS to a novel environment, with about 50% of copepods lost prior to discharge of ballast water. It appears that population loss caused the attenuation of OTUs in final samples. Therefore, our findings highlight that events that occur prior to introduction may influence genetic diversity of newly introduced populations, which, in turn, could affect subsequent establishment success.

## Conflict of Interest

None declared.

## Data Accessibility

OTUs and their matching accession numbers for each sample: Dryad doi: 10.5061/dryad.77sr0.

## Supporting information


**Figure S1.** Protocol for analysis of 19 ballast water samples collected during three Atlantic voyages.

**Figure S2.** Neighbor‐joining tree for all OTUs recovered from voyage one.

**Figure S3.** Neighbor‐joining tree for all OTUs recovered from voyage two.

**Figure S4.** Neighbor‐joining tree for all OTUs recovered from voyage three.

**Figure S5.** Sample‐based rarefaction curves from the initial (red lines), middle (green), and final (blue) sampling and 95% confidence intervals (dashed lines).

**Table S1.** Environmental characteristics (temperature, pH, dissolved oxygen (D.O.), and salinity) of three ballast water (A, B, and C) samples obtained at the initial (int), middle (mid), and final (fin) day during three voyages of a vessel transiting between Canada and Brazil.
**Table S2.** Standard ANOVA table for randomized block design based on the number of OTUs/sequences recovered from all voyages.
