# Supplementary figures and images for: Population attenuation in zooplankton communities during transoceanic transfer in ballast water
Source: Ecol Evol. 2016 Aug 2;6(17):6170–7. doi: 10.1002/ece3.2349 (PMC5016640; doi:10.1002/ece3.2349)

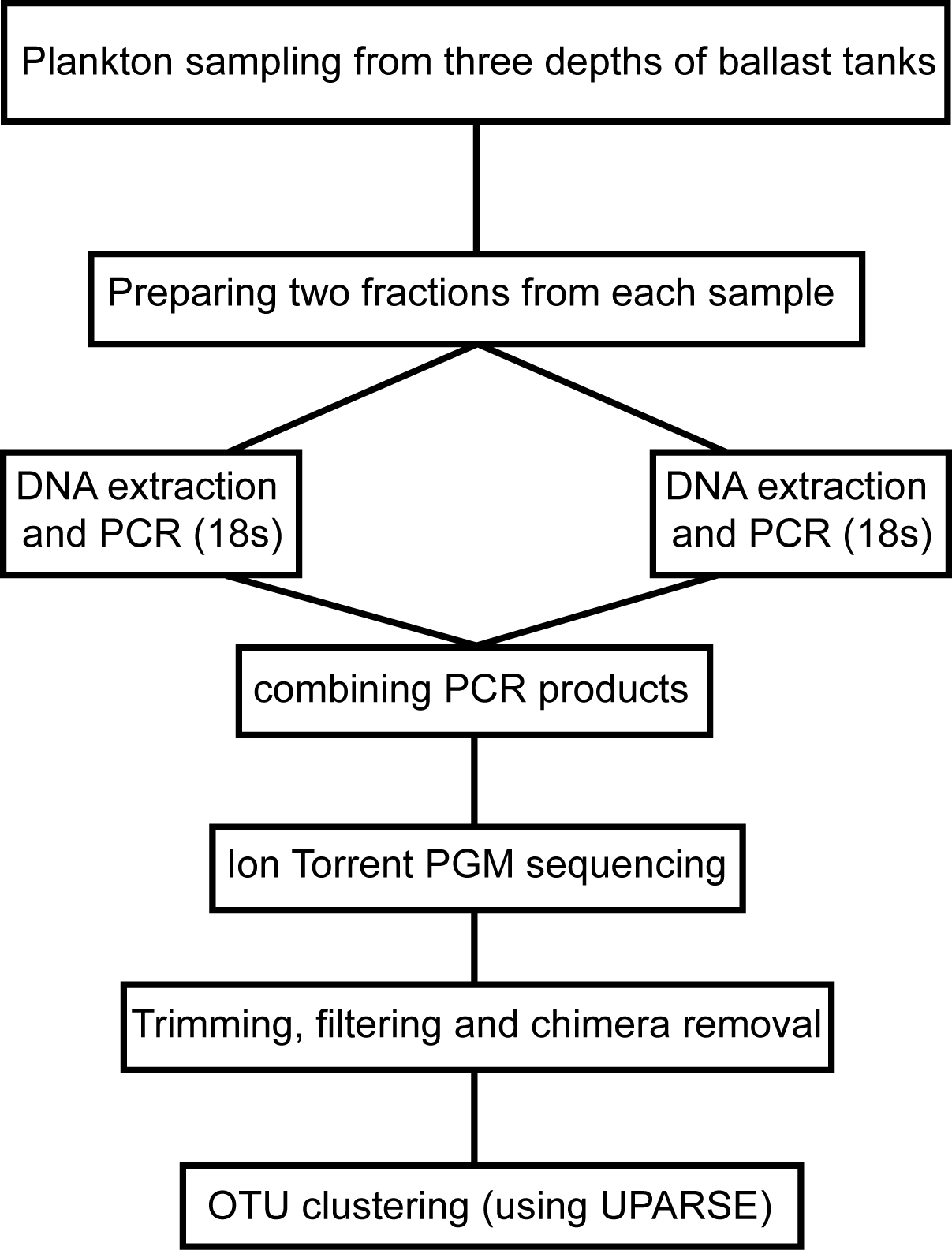

Supplement: Supplementary file 1 — Figure S1. Protocol for analysis of 19 ballast water samples collected during three Atlantic voyages. [file ECE3-6-6170-s001.tif]

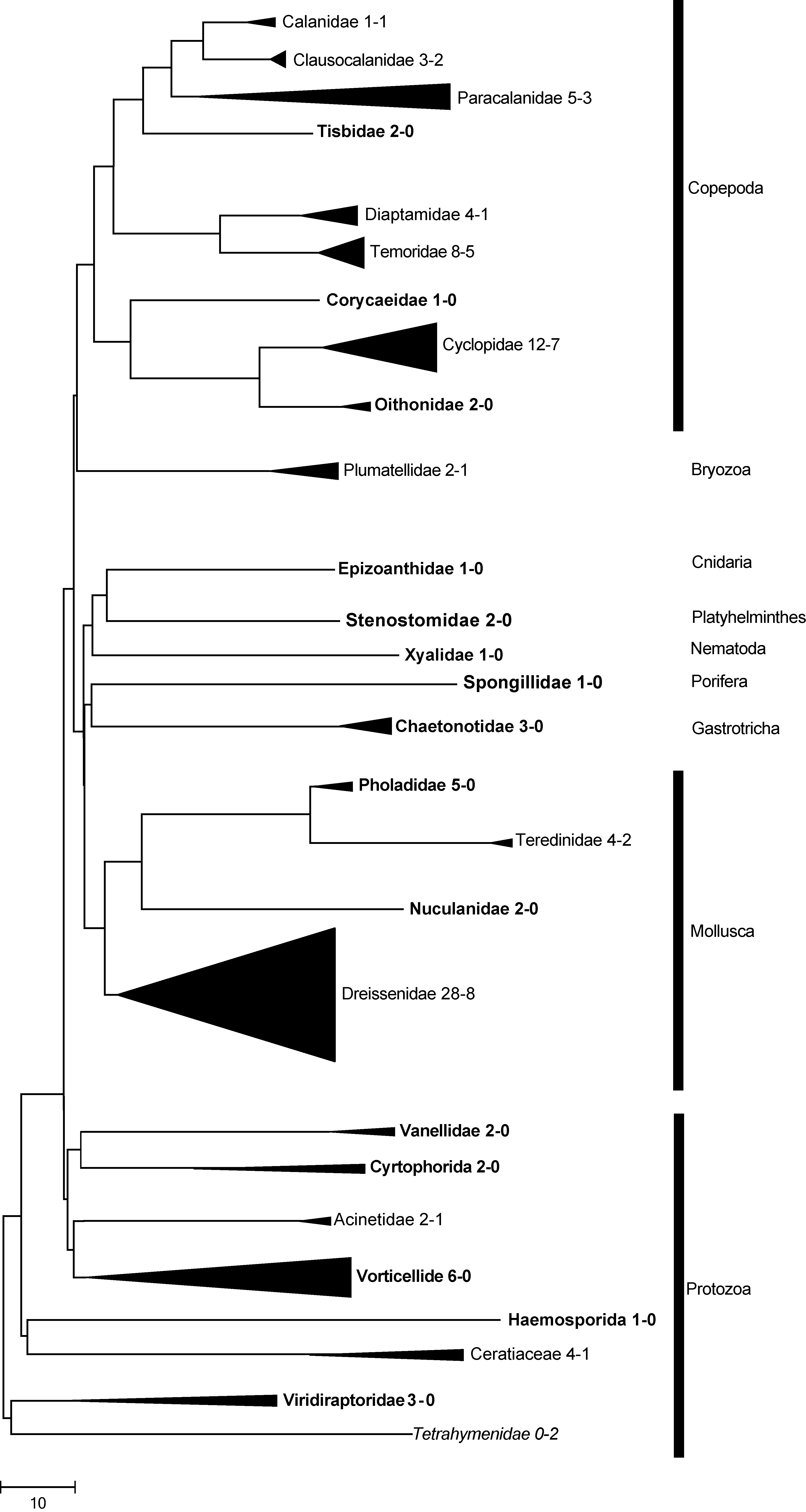

Supplement: Supplementary file 2 — Figure S2. Neighbor‐joining tree for all OTUs recovered from voyage one. [file ECE3-6-6170-s002.tif]

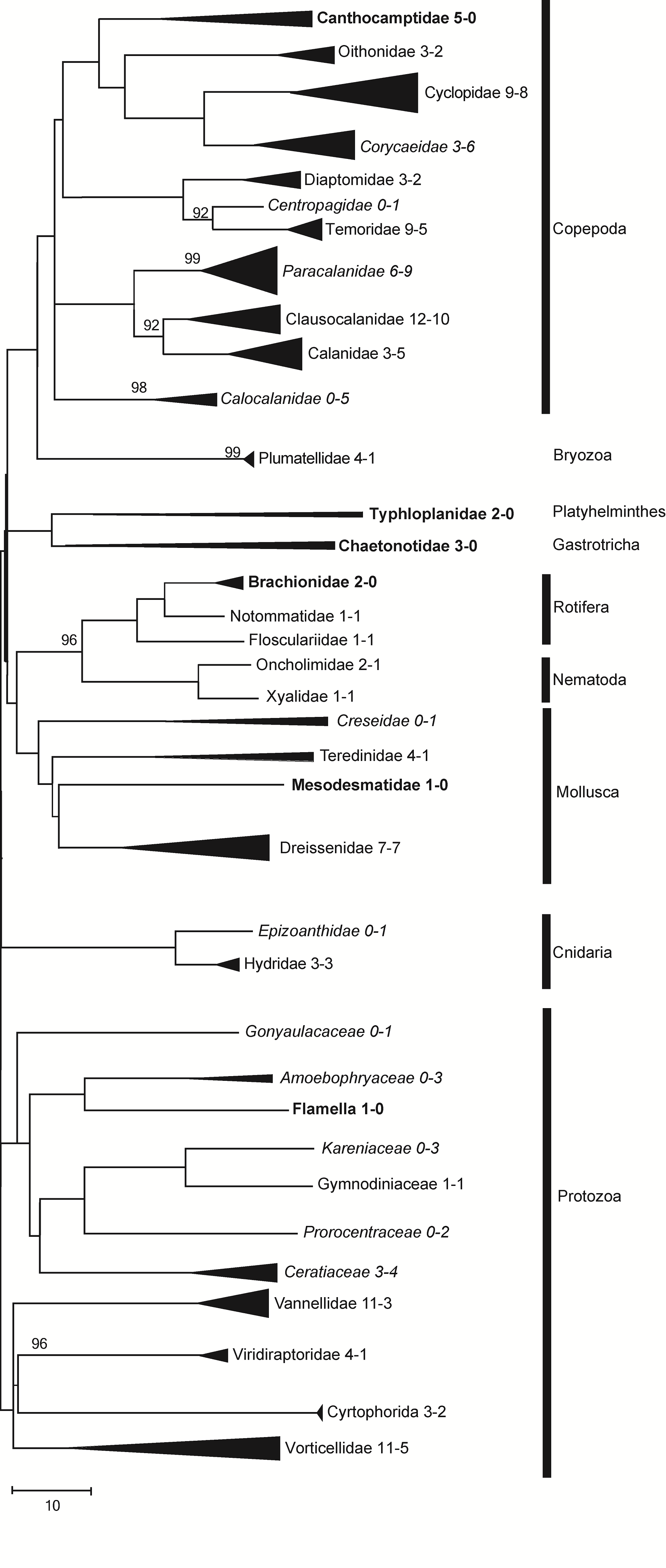

Supplement: Supplementary file 3 — Figure S3. Neighbor‐joining tree for all OTUs recovered from voyage two. [file ECE3-6-6170-s003.tif]

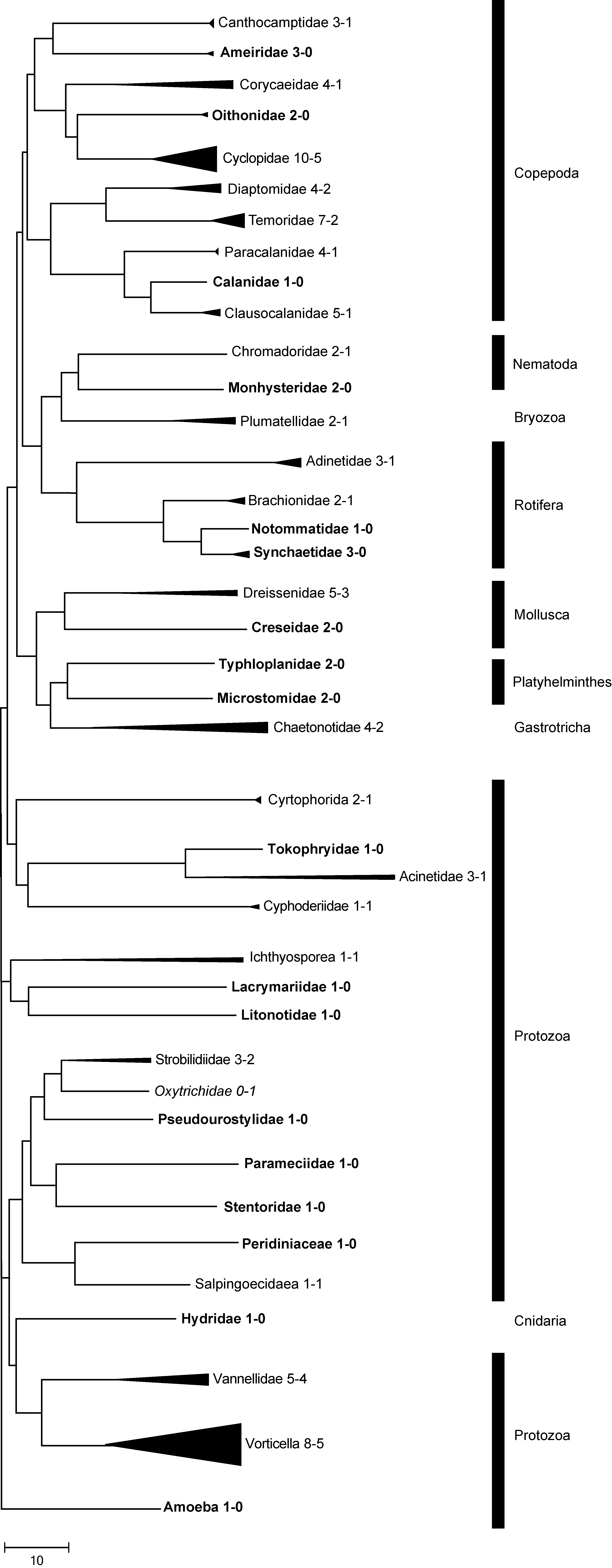

Supplement: Supplementary file 4 — Figure S4. Neighbor‐joining tree for all OTUs recovered from voyage three. [file ECE3-6-6170-s004.tif]

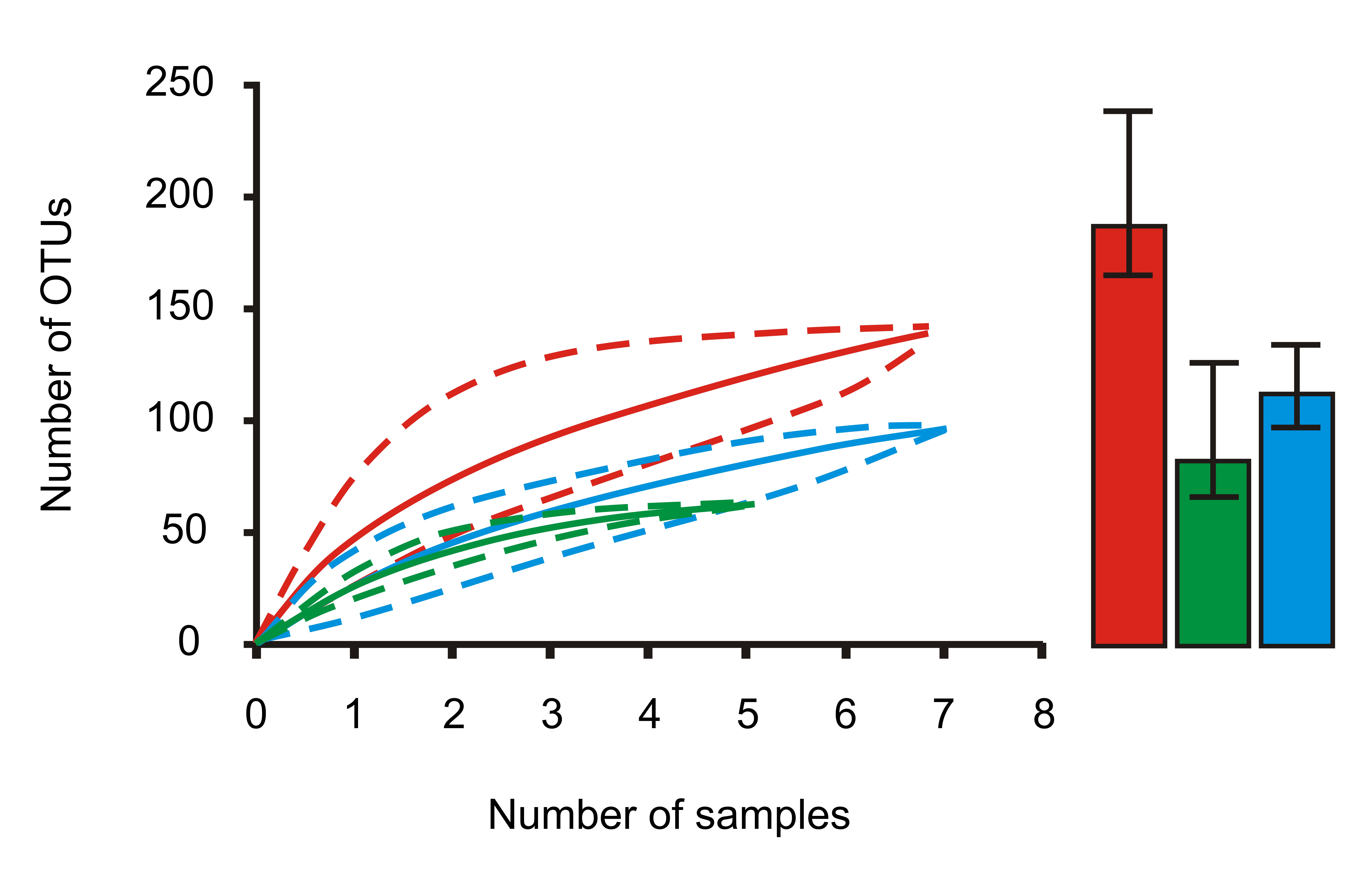

Supplement: Supplementary file 5 — Figure S5. Sample‐based rarefaction curves from the initial (red lines), middle (green), and final (blue) sampling and 95% confidence intervals (dashed lines). [file ECE3-6-6170-s005.jpg]
