# Supplementary material for: Population attenuation in zooplankton communities during transoceanic transfer in ballast water
Source: Ecol Evol. 2016 Aug 2;6(17):6170–7. doi: 10.1002/ece3.2349 (PMC5016640; doi:10.1002/ece3.2349)
Supplement: Supplementary file 6 — Table S1. Environmental characteristics (temperature, pH, dissolved oxygen (D.O.), and salinity) of three ballast water (A, B, and C) samples obtained at the initial (int), middle (mid), and final (fin) day during three voyages of a vessel transiting between Canada and Brazil. Table S2. Standard ANOVA table for randomized block design based on the number of OTUs/sequences recovered from all voyages. [file ECE3-6-6170-s006.doc]

**Supporting information**

**Table S1.** Environmental characteristics (temperature, pH, dissolved oxygen (D.O.), and salinity) of three ballast water (A, B, and C) samples obtained at the initial (int), middle (mid), and final (fin) day during three voyages of a vessel transiting between Canada and Brazil.

| Ballast tank | Sample (date) | Temperature (°C) | pH | D.O. % | Salinity (ppt) |
| --- | --- | --- | --- | --- | --- |
| 1A | int (03-06-12) | 26.5 | 8.0 | 37.3 | 0.1 |
|  | mid (07-06-12) | 19.4 | 8.4 | 59.2 | 3.3 |
|  | fin (11-06-12) | 26.2 | 8.3 | 77.0 | 0.3 |
| 1B | int (04-06-12) | 22.1 | 8.2 | 69.4 | 0.1 |
|  | mid (07-06-12) | 18.7 | 8.3 | 70.6 | 2.8 |
|  | fin (11-06-12) | 26.2 | 8.2 | 77.8 | 0.3 |
| 2A | int (17-09-12) | 10.2 | 8.1 | 19.2 | 13.8 |
|  | mid (20-09-12) | 11.5 | 7.9 | 35.0 | 17.2 |
|  | fin (24-09-12) | 25.5 | 7.5 | 22.0 | 17.4 |
| 2B | int (17-09-12) | 10.3 | 8.1 | 19.7 | 23.1 |
|  | mid (20-09-12) | 11.4 | 7.9 | 34.6 | 18.4 |
|  | fin (24-09-12) | 26.1 | 7.7 | 24.0 | 18.75 |
| 2C | int (17-09-12) | 7.7 | 8.1 | 19.0 | 20.2 |
|  | mid (20-09-12) | 11.4 | 7.8 | 58.0 | 20.4 |
|  | fin (24-09-12) | 24.3 | 7.5 | 22.0 | 20.8 |
| 3A | int (20-10-12) | 11.7 | 8.3 | 23.9 | 0.1 |
|  | fin (01-11-12) | 6.2 | 7.0* | 72.8 | 0.4 |
| 3B | int (18-10-12) | 11.3 | 8.4 | 55.2 | 0.1 |
|  | fin (01-11-12) | 6.6 | 7.0* | 73.1 | 0.6 |

*Samples were measured using test strips.

**Table S2.** Standard ANOVA table for randomized block design based on the number of OTUs/sequences recovered from all voyages.

| Source | Type IIWE sum of squares | df | Mean square | F | Sig. |
| --- | --- | --- | --- | --- | --- |
| Block (tank) | 398.39 | 6 | 66.40 | 0.83 | 0.574 |
| Time (# of OTUs) | 2431.79 | 2 | 1215.89 | 15.17 | 0.001 |
| Error | 801.38 | 10 | 80.14 |  |  |
| Corrected total | 3656.00 | 18 |  |  |  |
|  |  |  |  |  |  |
| Block (tank) | 3206685.76 | 6 | 534447.63 | 4.80 | 0.015 |
| Time (# of sequences) | 263868.78 | 2 | 131934.39 | 1.19 | 0.345 |
| Error | 1112255.72 | 10 | 111225.57 |  |  |
| Corrected total | 4592777.68 | 18 |  |  |  |
